# Sex steroid hormones and risk of breast cancer: a two-sample Mendelian randomization study

**DOI:** 10.1186/s13058-022-01553-9

**Published:** 2022-10-08

**Authors:** Aayah Nounu, Siddhartha P. Kar, Caroline L. Relton, Rebecca C. Richmond

**Affiliations:** MRC Integrative Epidemiology Unit, Bristol Medical School, University of Bristol, Bristol, UK

**Keywords:** Sex steroid hormones, Breast cancer, Mendelian randomization

## Abstract

**Background:**

Breast cancer (BC) has the highest cancer incidence and mortality in women worldwide. Observational epidemiological studies suggest a positive association between testosterone, estradiol, dehydroepiandrosterone sulphate (DHEAS) and other sex steroid hormones with postmenopausal BC. We used a two-sample Mendelian randomization analysis to investigate this association.

**Methods:**

Genetic instruments for nine sex steroid hormones and sex hormone-binding globulin (SHBG) were obtained from genome-wide association studies (GWAS) of UK Biobank (total testosterone (TT) *N*: 230,454, bioavailable testosterone (BT) *N*: 188,507 and SHBG *N*: 189,473), The United Kingdom Household Longitudinal Study (DHEAS *N*: 9722), the LIFE-Adult and LIFE-Heart cohorts (estradiol *N*: 2607, androstenedione *N*: 711, aldosterone *N*: 685, progesterone *N*: 1259 and 17-hydroxyprogesterone *N*: 711) and the CORtisol NETwork (CORNET) consortium (cortisol *N*: 25,314). Outcome GWAS summary statistics were obtained from the Breast Cancer Association Consortium (BCAC) for overall BC risk (*N*: 122,977 cases and 105,974 controls) and subtype-specific analyses.

**Results:**

We found that a standard deviation (SD) increase in TT, BT and estradiol increased the risk of overall BC (OR 1.14, 95% CI 1.09–1.21, OR 1.19, 95% CI 1.07–1.33 and OR 1.03, 95% CI 1.01–1.06, respectively) and ER + BC (OR 1.19, 95% CI 1.12–1.27, OR 1.25, 95% CI 1.11–1.40 and OR 1.06, 95% CI 1.03–1.09, respectively). An SD increase in DHEAS also increased ER + BC risk (OR 1.09, 95% CI 1.03–1.16). Subtype-specific analyses showed similar associations with ER+ expressing subtypes: luminal A-like BC, luminal B-like BC and luminal B/HER2-negative-like BC.

**Conclusions:**

TT, BT, DHEAS and estradiol increase the risk of ER+ type BCs similar to observational studies. Understanding the role of sex steroid hormones in BC risk, particularly subtype-specific risks, highlights the potential importance of attempts to modify and/or monitor hormone levels in order to prevent BC.

**Supplementary Information:**

The online version contains supplementary material available at 10.1186/s13058-022-01553-9.

## Introduction

Breast cancer (BC) is the most common cancer in women worldwide and is the leading cause of cancer mortality in females [1]. Early menarche and a later age at menopause have been shown to be associated with an increased risk of breast cancer [2]. Furthermore, a study conducted on postmenopausal women showed that a higher number of lifetime cumulative menstrual cycles increased BC risk [3]. Taken together, susceptibility to BC appears to be associated with ovarian hormones related to the menstrual cycle, although the biological basis for this is still not understood [4].

The association of oral contraceptive use and hormone replacement therapy (HRT) with BC risk provides further evidence for the role of ovarian hormones in BC. A systematic review that included 44 BC studies showed that oral contraceptive use increased the risk of BC [5]. A large-scale meta-analysis combining case–control data from 58 studies found that HRT use was associated with an increased risk of BC within 4 years of current use, with the increasing risk associated with a longer duration of current use [6].

Analyses looking specifically at blood levels of nine sex steroid hormones and BC risk concur with evidence surrounding factors associated with the menstrual cycle, oral contraceptive and HRT use with BC risk. A pooled analysis of nine prospective studies on 663 BC cases and 1765 controls found that increasing concentrations of oestrone, androstenedione, dehydroepiandrosterone (DHEA), dehydroepiandrosterone sulphate (DHEAS) and testosterone were associated with increased risk of BC in postmenopausal women [7]. Whilst most of these associations were thought to be due to the conversion of androgens (DHEA, DHEAS, testosterone and androstenedione [8, 9]) to estradiol, these associations remained even after adjustment for circulating estradiol levels [7, 10]. Androgen receptors have been shown to increase proliferation when expressed in triple-negative breast cancer (TNBC), further providing evidence for the role of androgens in BC risk independent of estradiol [11]. Positive associations with premenopausal BC were also found for estradiol, androstenedione, DHEAS and testosterone in a pooled analysis of seven prospective studies including 767 women with BC and 1699 controls [12]; however, a much larger study conducted in UK Biobank among 30,565 premenopausal women and 133,294 postmenopausal women found that testosterone and sex hormone-binding globulin (SHBG) increased and decreased BC risk in postmenopausal women, respectively, but did not influence premenopausal BC risk [13].

**Fig. 1:**
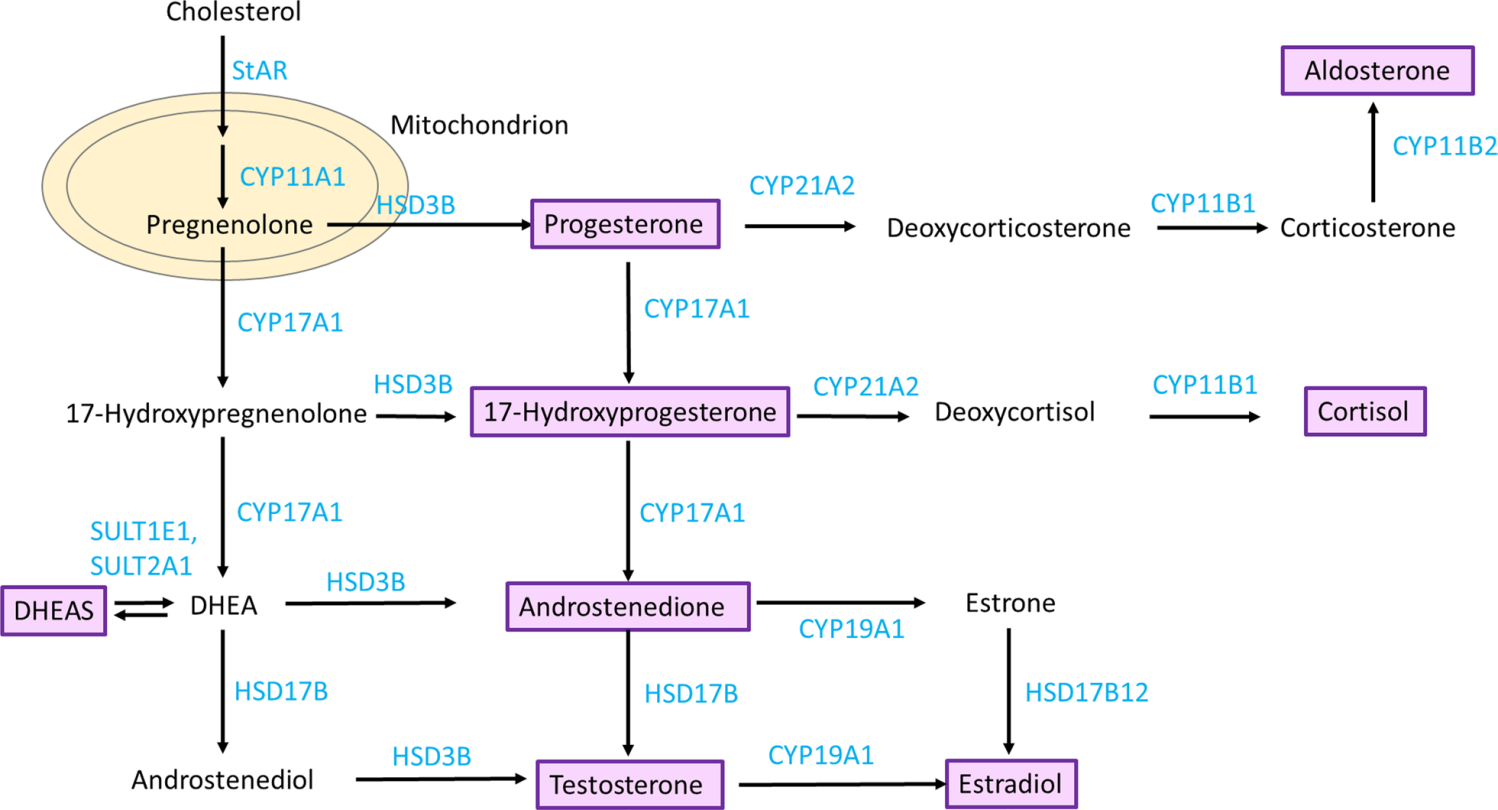
Sex steroid hormone metabolism pathway. Metabolites/hormones are displayed in black text and the enzymes that catalyse the reaction are in blue text. The hormones that are investigated in this analysis are shown in purple boxes. This diagram was adapted from Pott et al*.* [37]

Common metabolic pathways may underlie the relationship between sex hormones and BC risk. They are all produced from cholesterol and are synthesized in the gonads, adrenal cortex and placenta [14]. Cholesterol is first transported into the mitochondrion and converted to pregnenolone—the precursor for all sex hormones (Fig. 1) [15–17]. Whilst approximately half of the testosterone originates from the adrenal glands and the ovaries, the remainder is derived from the conversion of proandrogens (DHEA, DHEAS and androstenedione) in the periphery [18]. In postmenopausal women, the primary source of estradiol is from the conversion of androgens [19].

Much of the current evidence surrounding sex hormones and BC risk comes from observational epidemiological studies. However, these studies are prone to confounding, selection bias and other biases [20, 21]. The most reliable method for evaluating the effects of exogenous sex hormones on BC risk is through conducting randomized controlled trials (RCTs), but these are time-consuming and costly [22], especially in the case of primary prevention trials of cancer. For this reason, other approaches to causal inference such as Mendelian randomization (MR) can be used to provide evidence for or against the role of sex hormones. MR uses genetic variants that predict changes in exposures (e.g. hormone levels) and assesses their effect on outcome (e.g. BC) [21, 23, 24]. MR is analogous to an RCT as genetic variants are randomly allocated at conception, similar to random allocation of intervention at the start of a trial [25, 26], and fixed thereafter. This reduces the impact of confounding encountered in observational epidemiology [25].

Previous MR studies have been carried out looking at the effect of testosterone (total and bioavailable) and sex hormone-binding globulin (SHBG) on overall, ER+ and ER–BC risk [27, 28]. In this two-sample MR study, we expanded the analysis to include seven other sex steroid hormones as well as investigating the effect of the hormones on subtype-specific BC risk (luminal A-like BC, luminal B-like BC, luminal B/HER2-negative-like BC, HER2-enriched-like BC, TNBC and *BRCA1* mutated TNBC).

## Materials and methods

### Two-sample MR

To investigate the effect of sex hormone levels on BC risk, we applied a two-sample MR approach [29]. Firstly, genetic instruments to proxy for nine hormones and SHBG were obtained from GWAS summary statistics (sample 1). These were then integrated with BC risk genetic association effect estimates from published GWAS results (sample 2). We adopted a two-sample MR as opposed to a one-sample MR study because the risk of the winners’ curse that happens in a one-sample MR study is unlikely to happen in a two-sample MR. Furthermore, in the presence of weak instruments, bias in a two-sample MR is towards the null, whereas in one-sample MR it is biased towards the confounded multivariable regression result [29].

### Genetic predictors for sex hormones

Single nucleotide polymorphisms (SNPs) were extracted from summary data for total testosterone (TT), bioavailable testosterone (BT), DHEAS, estradiol, cortisol, androstenedione, aldosterone, progesterone, 17-hydroxyprogesterone (17OHP) and SHBG. The threshold for SNP selection was *P* < 5 × 10^–8^. When no SNPs were found at this association, the threshold was relaxed to *P* < 5 × 10^–7^, as was the case for estradiol, aldosterone and androstenedione (Table 2).

SNPs predicting levels of TT, BT and SHBG were obtained from publicly available summary statistics provided by Ruth et al*.* using UK Biobank data [27], which consists of phenotype and biological samples collected from around 500,000 individuals across Great Britain [30]. Testosterone and SHBG levels were measured (nmol/L) using a one-step competitive analysis and two-step sandwich immunoassay analysis, respectively, on a Beckman Coulter UniCel Dxl 800 in 230,454 and 189,473 participants, respectively [27]. BT (nmol/L; female *N*: 188,507) was calculated from TT and albumin, which was also measured by BCG analysis on a Beckman Coulter AU5800 (g/L). Genotyping and imputation (using the Haplotype Reference Consortium (HRC) and 1000 Genomes) and quality control (removal of SNPs with MAF ≤ 0.01) filtering resulted in 16,580,850–16,585,865 SNPs for the three measures (Table 2) [27]. Measures were subjected to inverse normal transformation of rank before being taken forward in a sex-stratified GWAS study [27].

Estradiol has also been measured in UK Biobank using a Beckman Coulter DXI 800 with a detection range between 73 and 17,621 pmol/L [31]. However, since the majority of females (162,691/273,455 (59.49%) [32], mean age 56.35 years [33]) enrolled in the UK Biobank were postmenopausal, levels of estradiol were below the limit of detection for 75% of these women [27, 34]. For this reason, we used summary data for estradiol from the LIFE-Adult and LIFE-Heart cohorts. The LIFE-Adult cohort consists of a random selection of 10,000 participants from Leipzig, Germany. Conversely, 7000 participants were chosen for the LIFE-Heart study based on having suspected or confirmed coronary artery disease. In contrast to UK Biobank, estradiol measurements in the LIFE-Adult and LIFE-Heart cohort (total *N*: 2607) were measured using an electrochemiluminescence immunoassay (ECLIA) with a detection limit of 18.4 pmol/L [35] and liquid chromatography-tandem mass spectrometry (LC–MS/MS) with a lower detection limit of 37 pmol/L, respectively [36]. For this reason, we used these two cohorts to obtain genetic instruments to proxy for estradiol levels. The mean age of women in the LIFE-Adult and LIFE-Heart cohorts was 59.4 and 64.8 years, respectively [37]. Imputation in this GWAS was performed using 1000 Genomes Phase 3 as the reference panel. Estradiol measurements were log-transformed prior to analysis, and so SNP associations represent a log-transformed unit increase (pmol/L) in levels (Table 2) [37].

Summary data were also obtained for the hormones androstenedione, aldosterone and 17-OHP, which were measured in females in LIFE-Heart only (*N* = 711, *N* = 685 and *N* = 711, respectively) (Table 2) using LC–MS/MS. Progesterone was measured in females in both LIFE-Heart and LIFE-Adult using LC–MS/MS (*N* = 1259). These GWASs were imputed using the 1000 Genomes reference panel with further information in Table 2. Steroid hormone measurements were log-transformed prior to analysis and so SNP associations represent a log-transformed unit increase (nmol/L or pmol/L) in hormone levels (Table 2) [37].

We obtained summary statistics for DHEAS associations from Prins et al*.* which included DHEAS measures for 9722 participants (4308 males and 5414 females) obtained from the United Kingdom Household Longitudinal Study—a longitudinal survey across the UK (England, Wales, Scotland and Northern Ireland) consisting of 40,000 households [38]. DHEAS was measured (µmol/L) in serum samples using a competitive immunoassay on the Roche E module analyser, and measurements were log-transformed and adjusted for age and sex; thereby, SNPs represented a log-transformed unit (µmol/L) increase in DHEAS levels [38]. Imputation was performed using the UK10K project and 1000 Genomes phase 3 panels in this GWAS (Table 2) [38].

We obtained summary statistics for cortisol from the CORtisol NETwork (CORNET) consortium that meta-analysed GWAS statistics from 17 population-based cohorts of a European background including 25,314 individuals (36.27% men and 63.73% women) [39]. Cortisol was measured using immunoassays in blood samples for all studies except TwinsUK which used liquid chromatography-mass spectrometry. The studies performed linear regressions on z-scores of log-transformed morning plasma cortisol and were also adjusted for sex, age and genetic ancestry. Imputation was performed using the Haplotype Reference Consortium and 1000 Genomes Phase 3 panels in this GWAS, with SNPs representing a standard deviation (SD) increase in cortisol levels (Table 2) [39].

For estradiol, DHEAS, progesterone, 17-OHP, aldosterone and androstenedione, we converted log-transformed units to a standard deviation scale so that results would represent an SD increase in hormone levels. For hormones with reported median and interquartile (IQR) ranges, these were transformed to the log scale, and the SD was calculated using the method presented by Wan et al*.* [40]. DHEAS study characteristics reported mean, maximum and minimum values, which were transformed to the log scale and the SD was calculated using the formula: (maximum–minimum)/4. When the hormone was measured in more than one study (estradiol, DHEAS and progesterone), a combined SD was calculated using the formula from the Cochrane Handbook (Sect. 6.5.2.10) [41].

To adjust for multiple testing, we applied a genome-wide significance threshold for SNP associations with metabolites (*P* value ≤ 5 × 10^–8^). When no SNPs were available at this cut-off, we relaxed the threshold to a *P* value ≤ 5 × 10^–7^ (Table 2). We also chose to include independent SNPs to avoid multi-collinearity and therefore carried out linkage disequilibrium (LD) clumping at an *R*^2^ < 0.001 so that only the SNP most strongly associated with the hormone within a 10,000 kb window was taken forward in the analysis.

We calculated the variance explained as well as the F statistic to assess whether the identified SNPs may be weak instruments. When weak instruments are used in a two-sample MR analysis, the estimate obtained tends to be biased towards the null [42]. The F statistic helps to determine the strength of the bias, with lower F statistics indicating a greater bias towards the null [43]. Power calculations were conducted using the mRnd online calculator to identify the effect size (odds ratio, OR) in both directions that could be detected based on the variance explained by the instruments and the sample sizes available [44]. Due to the absence of effect allele frequencies for the DHEAS GWAS, the variance explained, F statistic and power calculations could not be calculated for this hormone.

### Genetic associations for breast cancer

#### Breast cancer risk

Genetic association summary statistics for BC risk were obtained from the Breast Cancer Association Consortium (BCAC) (consisting of 68 studies combined together) as well as the Discovery, Biology and Risk of Inherited Variants in Breast Cancer Consortium (DRIVE) [45]. This study includes 122,977 BC cases and 105,974 controls and when stratified based on oestrogen receptor (ER) expression, there were 69,501 ER + BC cases and 21,468 ER–BC cases (Table 1). Genotyping was carried out using both the iCOGS array or the OncoArray with imputation (using the version 3 release of the 1000 Genomes Project data set as a reference panel) to obtain data on 10,680,257 SNPs and the results were combined using a fixed-effect meta-analysis [45].

**Table 1:** Outcome data sets

Breast cancer subtype	*N* Case	*N* controls	Year	References
Overall BC risk	122,977	105,974	2017	[45]
ER + BC risk	69,501	105,974	2017	[45]
ER–BC risk	21,468	105,974	2017	[45]
Luminal A BC risk	7325	20,815	2020	[46]
Luminal B BC risk	1682	20,815	2020	[46]
Luminal B and HER2 negative	1779	20,815	2020	[46]
HER2 enriched	718	20,815	2020	[46]
Triple negative	2006	20,815	2020	[46]
*BRCA1* mutated triple negative	18,016	100,971	2020	[46]

#### Breast cancer risk subtypes

To further identify subtype-specific effects of hormones on BC risk, we also tested their association with six subtypes of BC. Data from 118,474 cases and 96,201 controls were previously analysed from 82 studies from BCAC to obtain summary statistics associations for five subtypes of BC. These subtypes are defined by expression of ER, progesterone receptor (PR), human epidermal growth factor receptor 2 (HER2) and cancer grade: luminal A-like (ER+, and/or PR+, HER2- and grades 1/2; 7325 cases and 20,815 controls), luminal B-like (ER+ and/or PR+, HER2+; 1682 cases and 20,815 controls), luminal B/HER2-negative-like (ER+ and/or PR+, HER2-, grade 3; 1779 cases and 20,815 controls), HER2-enriched-like (ER-, PR-, HER2+; 718 cases and 20,815 controls) and TNBC (ER-, PR-, HER2-; 2006 and 20,815 controls) [46]. Furthermore, summary data from the Consortium of Investigators of Modifiers of BRCA1/2 (CIMBA) comprising 9414 cases with *BRCA1* mutation and 9494 controls with *BRCA1* mutation were also used in this analysis. Since the majority of *BRCA1*-mutated cancers were also triple-negative, we used summary statistics that meta-analysed associations of *BRCA1*-mutated cancers with TNBC (18,016 cases and 100,971 controls) (Table 1) [46].

### Statistical analysis

Analyses were carried out in R version 3.3.1 using the “Two-Sample MR” package [47], which allows data formatting, harmonization and application of MR methods in a semi-automated manner. This package automatically assigns the allele with a positive effect on the exposure as the effect allele, so that the effect allele predicts an increase in hormone levels. The SNPs used to proxy for the exposure are also extracted from the BC outcome data sets. Exposure and outcome summary statistics are then subject to allele harmonization to ensure that the effect allele in the exposure data set (hormone-increasing) is the same effect allele in the outcome data set, with effect allele frequencies used to assist in harmonizing palindromic SNPs.

In the presence of one SNP to proxy for hormone levels, Wald ratios (SNP-outcome estimate/SNP-exposure estimate) were used to calculate the change in log OR (risk analysis) per SD increase in hormone levels. When more than one SNP was present, an inverse variance weighted (IVW) method was applied, which is an average of the Wald ratios where the weight of the SNP contribution to the overall estimate is the inverse of the SNP effect on the outcome [48, 49]. The default of the two-sample MR package is a random effects IVW model; however, a fixed effects model is used when there is underdispersion in causal estimates between SNPs [47]. The fixed effects model assumes that there is no horizontal pleiotropy and that each SNP provides the same estimate, whereas the random effects model allows each SNP to have different means [50]. We performed the analysis for the risk of overall BC, ER + BC, ER–BC, luminal A-like BC, luminal B-like BC, luminal B/HER2-negative-like BC, HER2-enriched-like BC, TNBC and *BRCA1* mutated TNBC (Table 2).

**Table 2:** Exposure data sets

Hormone	Sample *N*	SNPs *N*	Year	Instruments N	Units	*P* value selection threshold	Variance explained (%)	F statistic	First author and reference
Total testosterone (TT)	230,454	16,580,850	2020	204	SD	5 × 10^–8^	2.36	27.29	Ruth [27]
Bioavailable testosterone (BT)	188,507	16,585,744	2020	131	SD	5 × 10^–8^	1.77	25.86	Ruth [27]
Sex hormone-binding globulin (SHBG)	189,473	16,585,865	2020	200	SD	5 × 10^–8^	3.35	32.81	Ruth [27]
Dehydroepiandrosterone sulphate (DHEAS)	9722	21,770,677	2017	4	Log-transformed unit (µmol/L) (converted to SD)	5 × 10^–8^	NA	NA	Prins [38]
Estradiol (E2)	2607	7,705,454	2019	2	Log-transformed unit (pmol/L) (converted to SD)	5 × 10^–7^	0.64	8.40	Pott [37]
Androstenedione (ANDRO)	711	8,799,744	2019	1	Log-transformed unit (nmol/L) (converted to SD)	5 × 10^–7^	0.44	3.10	Pott [37]
Aldosterone (ALDO)	685	8,806,555	2019	1	Log-transformed unit (pmol/L) (converted to SD)	5 × 10^–7^	0.55	3.79	Pott [37]
Cortisol	25,314	8,452,426	2021	1	SD	5 × 10^–8^	0.11	27.07	Crawford [39]
Progesterone (PROG)	1259	8,799,744	2019	3	Log-transformed unit (nmol/L) (converted to SD)	5 × 10^–8^	0.64	2.71	Pott [37]
17-hydroxyprogesterone (17OHP)	711	8,799,744	2019	1	Log-transformed unit (nmol/L) (converted to SD)	5 × 10^–8^	0.11	0.78	Pott [37]

The IVW method is prone to bias if one of the genetic instruments is invalid due to its association with another trait through an independent pathway (horizontal pleiotropy) [51]. For this reason, we also applied alternative MR methods that produce unbiased estimators even in the presence of some invalid genetic instruments. When more than two SNPs were present, we calculated a weighted median, weighted mode and an MR-Egger estimate [47, 52–54]. The weighted median approach allows a consistent estimate even if 50% of the information contributing to the overall estimate comes from invalid genetic instruments [53]. The weighted mode estimate may also be used even when the majority of the SNPs are invalid instruments so long as the SNPs that form a cluster of homogenous results are valid [48, 52]. Finally, we adopted an MR-Egger analysis to evaluate evidence for the presence of horizontal pleiotropy. This method is not constrained to pass through an effect size of 0; therefore, the y-intercept gives an indication of the presence of directional pleiotropy [51, 54]. We used an MR-Egger intercept with a *P* value below 0.05 to indicate the presence of directional pleiotropy that may be influencing the MR results. For hormones proxied by weak instruments, we conducted an MR robust adjusted profile score (MR RAPS), a method that provides robust inference when weak instruments are present [44].

Linkage disequilibrium score regression (LDSC) was used to assess the genetic correlation between TT and BT with estradiol using the settings advised in the software package LDSC (v1.0.1) [55]. We tested these hormones due to the direct conversion of testosterone to estradiol and therefore to identify whether SNP associations are shared between the two traits. Firstly, quality control was performed on the summary statistics to exclude variants with missing data, non-biallelic, strand-ambiguous alleles which could not be matched in the European ancestry 1000 Genomes reference panel, variants with imputation scores below 0.90 and rare variants with minor allele frequencies below 0.01. A score was calculated to reflect whether GWAS test statistics for variants correlates with nearby variants that are in high LD. A z statistic was generated for each variant in trait 1, and this was multiplied with the z statistic from trait 2. The product was regressed against the LD scores, and the resultant coefficient/slope was the genetic correlation statistic.

## Results

### Two-sample MR analysis of sex hormones and breast cancer risk

To investigate the effect of sex hormones on BC risk, we conducted an MR analysis of nine hormones and SHBG on overall, ER+ and ER–BC risk.

**Fig. 2:**
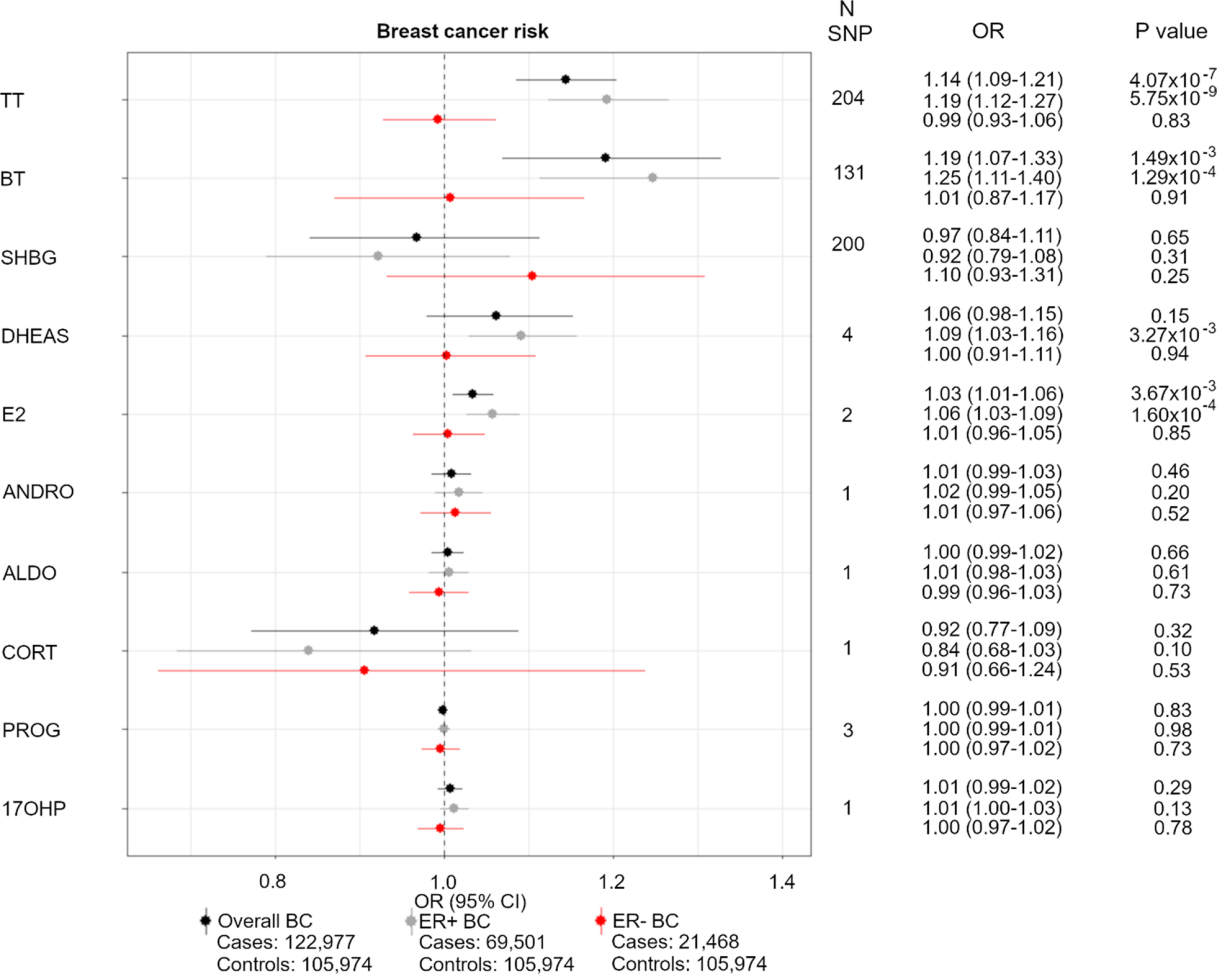
Forest plot showing the MR associations between sex hormones and overall, ER+ and ER–BC risk. IVW analysis was carried out to assess the association between an SD increase in total testosterone, bioavailable testosterone, SHBG, DHEAS, estradiol, androstenedione, aldosterone, cortisol, progesterone and 17-OHP on risk of incidence of overall BC (black), ER + BC (grey) and ER–BC (red). *OR* odds ratio, *TT* total testosterone, *BT* bioavailable testosterone, *SHBG* sex hormone-binding globulin, *DHEAS* dehydroepiandrosterone sulphate, *E2* estradiol, *ANDRO* androstenedione, *ALDO* aldosterone, *CORT* cortisol, *PROG* progesterone, *17OHP* 17-hydroxyprogesterone

After clumping at *R*^2^ < 0.001, we identified 204 and 131 SNPs to proxy for an SD increase in TT and BT at *P* ≤ 5 × 10^–8^, respectively (Additional file 1: Table S1). These SNPs explain 2.36% and 1.77% of the variance in the hormone levels and have an F statistic of 27.29 and 25.87, respectively (Additional file 1: Table S2). Using an IVW approach, we found that an SD increase in TT increased the risk of overall BC and ER + BC (OR 1.14, 95% CI 1.09–1.21 and OR 1.19, 95% CI 1.12–1.27, respectively) but had no effect on ER–BC (OR 0.99, 95% CI 0.93–1.06) (Fig. 2). We also found positive associations between TT and overall BC risk using the MR-Egger, weighted median and weighted mode methods (OR 1.22, 95% CI 1.10–1.34, OR 1.13, 95% CI 1.06–1.20 and OR 1.17, 95% CI 1.08–1.26, respectively) as well as a positive association with ER + BC risk (MR-Egger OR 1.29, 95% CI 1.16–1.45, weighted median OR 1.17, 95% CI 1.09–1.27 and weighted mode OR 1.23, 95% CI 1.13–1.35) (Additional file 1: Table S3). Furthermore, we conducted an MR-Egger intercept test but found no evidence of directional pleiotropy for overall BC risk and ER + BC risk (Additional file 1: Table S4).

We also found that an SD increase in BT increased the risk of overall and ER + BC (OR 1.19, 95% CI 1.07–1.33 and OR 1.25, 95% CI 1.11–1.40, respectively). Further positive associations were found using the weighted median approach (overall BC risk: 1.13, 95% CI 1.02–1.24 and ER + BC OR 1.23, 95% CI 1.10–1.38) and suggestive positive associations from the MR-Egger and weighted mode approaches (Additional file 1: Table S3).

We found two SNPs at *P* ≤ 5 × 10^–7^ to proxy for an SD increase in estradiol, which explained 0.64% of the variance with an F statistic of 8.40. Although the F statistic is low, one of the SNPs (rs2414098) is an intronic variant in the gene *CYP19A1* [56] which encodes the enzyme involved in the conversion of testosterone to estradiol [37]. For this reason, we continued with the MR analysis and found an increased risk of both overall BC risk and ER + BC (OR 1.03, 95% CI 1.01–1.06 and OR 1.06, 95% CI 1.03–1.09, respectively) but no effect on ER–BC risk (OR 1.01, 95% CI 0.96–1.05).

Due to the possibility of weak instrument bias, we also used the MR-RAPS method and found a positive association between an SD increase in estradiol with overall and ER + BC (OR 1.04, 95% CI 1.01–1.06 and OR 1.06, 95% CI 1.02–1.09, respectively). Since only two SNPs were used as proxies for estradiol, no other MR methods were used to test this association. We also calculated the genetic correlation between TT and BT with estradiol, but found no strong evidence of correlation (TT r_g:_ 0.25 (95% CI − 0.05–0.54) and BT r_g:_ 0.09, (95% CI − 0.13–0.31)) (Additional file 1: Table S5), indicating that the effect of estradiol on BC risk may be independent of testosterone levels.

Similar to TT, BT and estradiol, we found a positive association between an SD increase in DHEAS (proxied by 4 SNPs) and ER + BC risk (OR 1.09, 95% CI 1.03–1.16), with positive associations also observed using the weighted median, weighted mode and MR-Egger approaches (OR 1.08, 95% CI 1.04–1.13, OR 1.08, 95% CI 1.03–1.13, OR 1.07, 95% CI 0.97–1.18, respectively) (Additional file 1: Table S3).

We found little evidence of association between SHBG or cortisol levels and overall, ER+ and ER–BC risk. With regards to androstenedione, aldosterone, progesterone and 17-OHP, we also found little evidence of associations with overall, ER+ and ER–BC risk. However, we acknowledge that the genetic instruments used to proxy these hormones may be weak as demonstrated by low F statistics (0.78–3.79) [57] (Table 1). For this reason, we also carried out a weak instrument robust method—MR-RAPS, but still found little evidence of an association between the four hormones and overall, ER+ and ER–BC risk (Additional file 1: Table S3).

### Two-sample MR analysis of sex hormones and breast cancer subtype risk

To investigate the effect of sex hormones on the risk of specific BC subtypes, we conducted an MR analysis of the nine hormones and SHBG on luminal A-like BC, luminal B-like BC, luminal B/HER2-negative-like BC, HER2-enriched-like BC, TNBC and *BRCA1*-mutated TNBC. Details on the SNPs used as instruments in this analysis are found in Additional file 1: Tables S6 and S7.

**Fig. 3:**
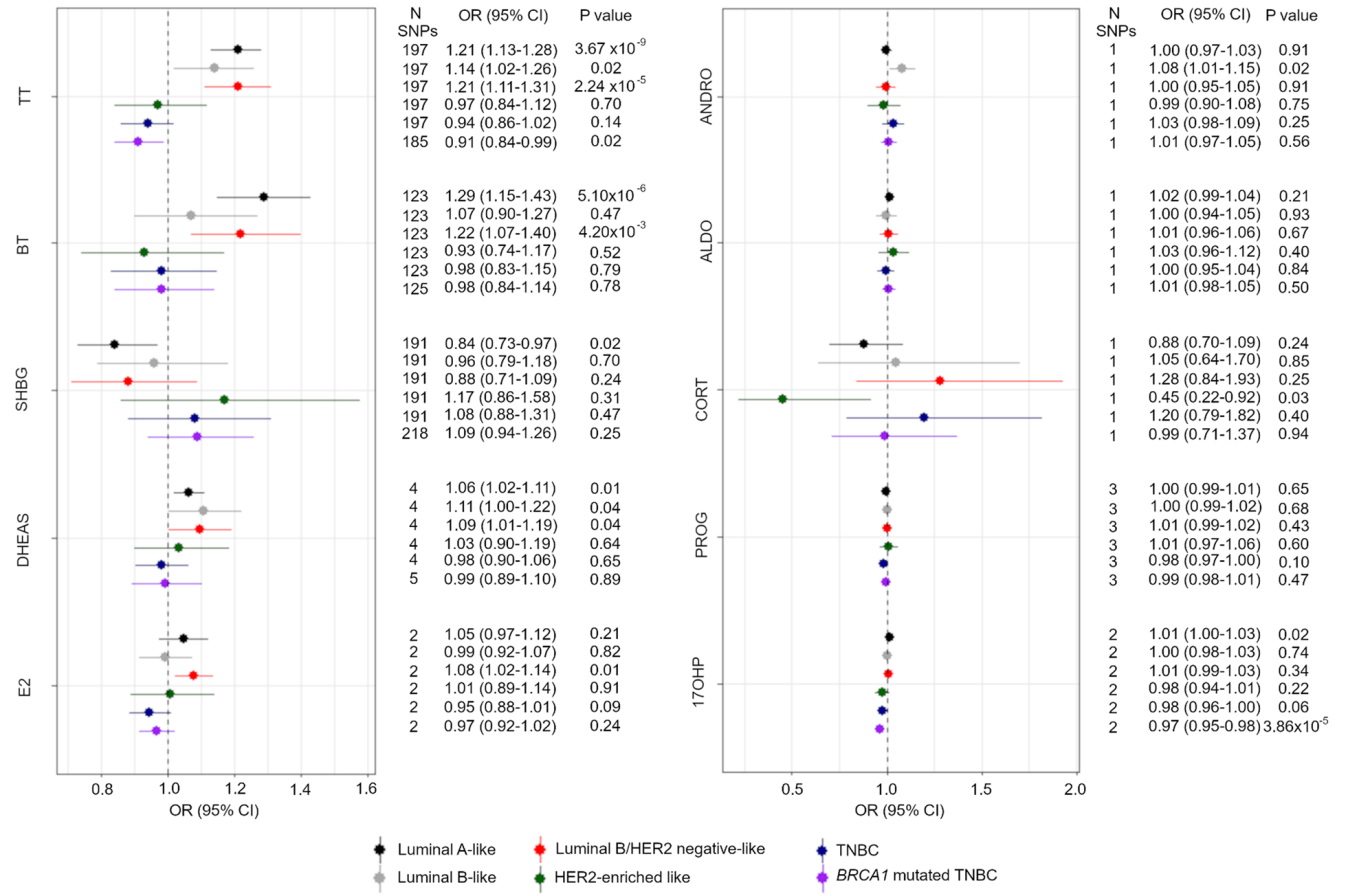
Forest plot showing the MR associations between sex hormones and risk of six BC subtypes. IVW analysis was carried out to assess the association between an SD increase in total testosterone, bioavailable testosterone, SHBG, DHEAS, estradiol, androstenedione, aldosterone, cortisol, progesterone and 17-OHP on risk of luminal A BC (black), luminal B (grey), luminal B and HER2-negative BC (red), HER2-enriched BC (green), triple-negative BC (blue) and *BRCA1* mutated triple-negative BC (purple). *OR* odds ratio, *TT* total testosterone, *BT* bioavailable testosterone, *SHBG* sex hormone-binding globulin, *SHBG adjusted* SHBG adjusted for BMI, *DHEAS* dehydroepiandrosterone sulphate, *E2* estradiol, *ANDRO* androstenedione, *ALDO* aldosterone, *CORT* cortisol, *PROG* progesterone, *17OHP* 17-hydroxyprogesterone, *TNBC* triple-negative BC

We found that an SD increase in TT levels increased the risk of luminal A-like BC, luminal B-like BC and luminal B/HER2-negative-like BC (OR 1.21, 95% CI 1.13–1.28, OR 1.14, 95% CI 1.02–1.26 and OR 1.21, 95% CI 1.11–1.31, respectively) (Fig. 3). Similar directions of association were found using the weighted median, weighted mode and MR-Egger approaches (Additional file 1: Table S3) with the MR-Egger intercept showing no evidence of directional pleiotropy (Additional file 1: Table S4). Conversely, an SD increase in TT was associated with a decreased risk of *BRCA1*-mutated TNBC (OR 0.91, 95% CI 0.84–0.99). We found positive associations between an SD increase in BT and luminal A-like BC and luminal B/HER2-negative-like BC risks (OR 1.29, 95% CI 1.15–1.43 and OR 1.22, 95% CI 1.07–1.40) with consistent directions of association found using the weighted median, weighted mode and MR-Egger approaches for the association with luminal A-like BC. However, only the MR-Egger approach showed a positive association for luminal B/HER2-negative-like BC risk (Additional file 1: Table S3). In contrast, the MR-Egger intercept showed little evidence of directional pleiotropy for any of these associations (Additional file 1: Table S4).

We found an inverse association between an SD increase in levels of SHBG and luminal A-like BC risk (OR 0.84, 95% CI 0.73–0.97); however, the weighted median, weighted mode and MR-Egger approaches showed little evidence of association (Additional file 1: Table S3).

Similar to TT and BT, we found that an SD increase in estradiol increased the risk of luminal B/HER2-negative-like BC (OR 1.08, 95% CI 1.02–1.14) and a possible inverse association with the more aggressive subtype of cancer (TNBC IVW OR 0.95, 95% CI 0.88–1.01) (Fig. 3). We also found similar associations using the MR-RAPS method (OR 1.08, 95% CI 1.01–1.15 and OR 0.94, 95% CI 0.89–1.00). Due to a limited number of SNPs, further MR methods could not be applied.

We also found positive associations between an SD increase in DHEAS and luminal A-like BC, luminal B-like BC and luminal B/HER2-negative-like BC (OR 1.06, 95% CI 1.02–1.11, OR 1.11, 95% CI 1.00–1.22 and OR 1.09, 95% CI 1.01–1.19, respectively) and found concurring results with the weighted median approach for all subtypes and suggestive positive associations for both the weighted mode and MR-Egger approaches (Additional file 1: Table S3). We saw little evidence of directional pleiotropy from the MR-Egger intercept (Additional file 1: Table S4).

With regards to cortisol, we found an inverse association between an SD increase in the hormone and HER2-enriched-like BC (OR 0.45, 95% CI 0.22–0.92). The F statistics and variance explained for the remaining four hormones (androstenedione, aldosterone, progesterone and 17-OHP) were suggestive of weak instruments and power calculations showed that we were underpowered to detect an effect for some of the BC subtypes (Additional file 1: Table S8). We found a positive association between androstenedione and luminal B-like BC using the IVW method (OR 1.08, 95% CI 1.01–1.15), but this effect was no longer observed using the weak instrument—robust MR-RAPS method (OR 1.02, 95% CI 0.99–1.05). The MR-RAPS method did suggest a possible association of androstenedione with TNBC (OR 1.08, 95% CI 1.00–1.17). We found little evidence of associations between aldosterone and progesterone with the risk of the six BC subtypes using both the IVW and MR-RAPS method. Finally, we observed a positive association between an SD increase in 17-OHP and luminal A-like BC (OR 1.01, 95% CI 1.00–1.03) and an inverse association with *BRCA1*-mutated TNBC (OR 0.97, 95% CI 0.95–0.98) using the IVW method. Both of these associations were also observed when using the MR-RAPS method (luminal A-like BC OR 1.01, 95% CI 1.00–1.03 and *BRCA1*-mutated TNBC OR 0.97, 95% CI 0.95–0.99).

## Discussion

In this study, we aimed to assess whether nine sex steroid hormones and SHBG affect BC risk using an MR framework. Overall, we found that an increase in TT, BT, estradiol and DHEAS was associated with overall BC and/or ER + BC.

The association of TT and BT with overall BC as well as ER + BC risk has been reported before using MR methods [27]. However, our study also investigated subtype-specific associations using MR in ER+ tumours luminal A-like, luminal B-like and luminal B/HER2-negative-like BC. Associations with ER+ tumours involving testosterone may be explained by two possible mechanisms—the first involves its conversion to estradiol [19] which binds to the ER and induces transcription of growth-positive genes and reduces expression of negative regulators of cell growth, therefore increasing breast cancer cell proliferation [58]. The second possible explanation for the association may be due to ER expression acting as a proxy for androgen receptor (AR) expression of which AR expression is positively correlated with ER expression in tumours [59, 60]. This is further supported by the finding that only 20–30% of ER–BCs express AR [61]. The literature suggests that resistance to ER therapies may be due to tumour adaptation towards androgen dependence and AR signalling instead, and it has been suggested that patients with ER+/AR+ tumours would most likely benefit from combination therapies targeting both receptors [62]. In order to try and untangle the mechanism through which testosterone acts in breast cancer, genetic association studies on tumour subtypes stratified based on AR expression and ER expression are required.

Our study demonstrated a relationship between estradiol and BC risk in an MR framework. An SD increase in estradiol increased the risk of overall BC and ER + BC as well as the ER + BC subtype luminal B/HER2-negative-like. We used summary statistics from Pott et al*.* to identify suitable genetic instruments for estradiol [37]. Despite the more sensitive methods for measuring E2, the average levels of the hormones detected for LIFE-Adult and LIFE-Heart were 18.4 pmol/L and 11.1 pmol/L, respectively[37], compared to ≥ 200 pmol/L in premenopausal women [37]. This may be because the average age of women in these studies was 59.4 and 64.8 years, respectively, which indicates that a large percentage of these cohorts may have been postmenopausal. Postmenopausal women no longer produce estradiol from the ovaries, and so production of this hormone is through the conversion of androgens to estradiol which occurs at the tissue of interest [19]. Therefore, estradiol production in postmenopausal women is localized and may have resulted in lower detection of estradiol in the blood.

These low levels of estradiol may explain why we only had two instruments to proxy for the hormone and why they only explained 0.64% of the variance, with a low F statistic indicating that these were weak instruments (8.40). However, one SNP (rs2414098) near *CYP19A1* shows evidence for a key biological role in affecting hormone levels, with suggestive evidence that it is associated with an increase in *CYP19A1* expression in breast tissue and would theoretically result in increased conversion of testosterone to estradiol. This plausible biological role of the SNP supports the results, despite the F statistic suggesting weak instruments. We also find no strong evidence of genetic correlation between TT and BT, indicating that the estradiol effect on BC risk may be independent of testosterone.

Similarly, an SD increase in DHEAS was associated with an increased risk of ER + BC, and subtype analysis showed positive associations with the three ER + BC subtypes: luminal A-like, luminal B-like and luminal B/HER2-negative-like BC. These results support positive associations found in observational studies between DHEAS levels and BC risk in postmenopausal women [7, 63].

While observational studies have shown generally consistent results with regards to sex hormones and postmenopausal BC risk, few studies have looked at the association with premenopausal BC. ER + BC is generally found in older and postmenopausal women, and ER−BC is generally found in younger premenopausal women [64]. Analysis of seven prospective studies found that doubling concentrations of estradiol, androstenedione, DHEAS and testosterone all increased the risk of premenopausal BC [12]. Unlike postmenopausal women, premenopausal women produce estradiol in the ovaries which then circulates in the blood [15]. The difficulty in trying to understand the relationship between estradiol and BC in premenopausal women is due to the much smaller sample sizes of cases in prospective cohorts as well as difficulty in accounting for the phase of the menstrual cycle which impacts measures of estradiol [15]. For this reason, the association between estradiol and premenopausal BC is still unclear [15]. It is important to note that the sex steroid hormone GWASs used in this study have mostly been conducted on older-aged women of which a large percentage are postmenopausal. Since ER–BC tends to occur more commonly in premenopausal women, instruments robustly predicting hormonal levels in premenopausal women need to be identified and used instead.

Whilst our study showed associations between testosterone, estradiol, DHEAS and cortisol with various BC subtypes’ risk in an MR framework, it is not without limitations. Firstly, the sample sizes of the GWAS from which some of our exposure instruments were derived were relatively small, and therefore the instruments used were weak, especially in the case of androstenedione, aldosterone, progesterone and 17-OHP. In the case of a two-sample MR setting, using weak instruments will bias the causal estimate towards the null [65] and may explain some of the null associations observed. The lack of genome-wide significant SNPs for androstenedione and aldosterone may have been due to the small sample sizes of the GWASs for these hormone measurements (*N* = 712 and *N* = 686, respectively). In addition, participants in the LIFE-Heart study were selected based on suspected or confirmed coronary artery disease, indicating possible selection bias. Furthermore, we derived genetic instruments for DHEAS and cortisol from mixed populations, due to much larger sample sizes than female-specific GWASs. This means that larger GWASs specifically in females need to be performed to identify stronger genetic instruments for these hormones before definitive conclusions on null associations can be made.

Further limitations include that our study also does not differentiate between exogenous sources of these hormones or endogenous, which is important for public health interventions such as advising for or against oral contraceptives and HRT use. We also acknowledge that the effect sizes observed in this study are small—the highest effect association was found between BT and luminal A BC risk (OR 1.29, 95% CI 1.15–1.43), indicating that increased hormone levels may slightly increase the risk of BC and that perhaps higher levels obtained through exogenous sources increase this risk further. Furthermore, MR investigates the lifetime effect of an exposure [23], whereas these drugs are often taken at specific time points or for certain durations. Therefore, it is difficult to identify the duration for which these drugs or exogenous sources of hormones could be affecting the risk of disease. Moreover, genetic instruments used in MR studies typically proxy a small amount of variation in the exposure [66]. For some exposures, larger variations may be required to detect an effect on an outcome which MR would otherwise show as null.

## Conclusions

Overall, our results suggest that increasing levels of testosterone, BT, estradiol and DHEAS may increase the risk of overall BC and/or ER+ (postmenopausal) BC risk, consistent with results from observational studies. For the remaining hormones, we found some suggestive associations but also acknowledge the possibility of weak instrument bias and the need for better genetic instruments. Our study provides new insights into the role of sex steroid hormones in BC risk using MR and may inform the eventual development of interventions aimed at BC prevention.

## Supplementary Information

**Additional file 1. S1**. Exposure instruments to proxy hormone levels in the overall breast cancer incidence MR analysis.** S2**. The strength of the instruments for each hormone including the variance explained and the F statistic.** S3**. Hormone and breast cancer incidence associations using the alternative MR methods: MR Egger, weighted median, weighted mode and MR-RAPS.** S4**. MR intercept calculations to identify evidence of pleiotropy.** S5**. Genetic correlation results between hormones.** S6**. Exposure instruments to proxy hormone levels in the subtype specific MR analysis.** S7**. Exposure instruments to proxy hormone levels in the BRCA1 mutated TNBC MR analysis.** S8**. Power calculations for each hormone and overall and subtype specific BC analysis.

## Data Availability

Summary statistics for total testosterone, bioavailable testosterone and SHBG are available at: https://www.ebi.ac.uk/gwas/publications/32042192. Summary statistics for cortisol are available at: https://datashare.ed.ac.uk/handle/10283/3836. Summary statistics for estradiol, androstenedione, aldosterone, progesterone and 17-hydroxyprogesterone are not publicly available to preserve confidentiality or because they were used under license but access to some of the data may be provided upon contact of the study’s corresponding author. Summary statistics for breast cancer incidence are available at http://bcac.ccge.medschl.cam.ac.uk/bcacdata/.

## Abbreviations

17OHP: 17-Hydroxyprogesterone
ALDO: Aldosterone
ANDRO: Androstenedione
AR: Androgen receptor
BC: Breast cancer
BCAC: Breast Cancer Association Consortium
BT: Bioavailable testosterone
CIMBA: Consortium of Investigators of Modifiers of BRCA1/2
CORNET: CORtisol NETwork
CORT: Cortisol
DHEA: Dehydroepiandrosterone
DHEAS: Dehydroepiandrosterone sulphate
DRIVE: Discovery, Biology and Risk of Inherited Variants in Breast Cancer Consortium
E2: Estradiol
ECLIA: Electrochemiluminescence immunoassay
ER: Oestrogen receptor
HER2: Human epidermal growth factor receptor 2
HR: Hazard ratio
HRT: Hormone replacement therapy
IQR: Interquartile
IVW: Inverse variance weighted
LD: Linkage disequilibrium
LDSC: Linkage disequilibrium score regression
LM-MS/MS: Liquid chromatography-tandem mass spectrometry
MR: Mendelian randomization
MR-RAPS: MR robust adjusted profile score
OR: Odds ratio
PR: Progesterone receptor
PROG: Progesterone
RCT: Randomized controlled trial
SD: Standard deviation
SHBG: Sex hormone-binding globulin
SNP: Single nucleotide polymorphism
TNBC: Triple-negative breast cancer
TT: Total testosterone
